# Mycobacterium bovis Requires P27 (LprG) To Arrest Phagosome Maturation and Replicate within Bovine Macrophages

**DOI:** 10.1128/IAI.00720-16

**Published:** 2017-02-23

**Authors:** Cristina Lourdes Vázquez, María Verónica Bianco, Federico Carlos Blanco, Marina Andrea Forrellad, Maximiliano Gabriel Gutierrez, Fabiana Bigi

**Affiliations:** a Instituto de Biotecnología, CICVyA-INTA, Nicolás Repetto y De Los Reseros, Buenos Aires, Argentina; b Host-Pathogen Interactions in Tuberculosis Laboratory, The Francis Crick Institute, London, United Kingdom; Weill Cornell Medical College

**Keywords:** LprG, Mycobacterium bovis, P27

## Abstract

Mycobacterium bovis causes tuberculosis in a wide variety of mammals, with strong tropism for cattle and eventually humans. P27, also called LprG, is among the proteins involved in the mechanisms of the virulence and persistence of M. bovis and Mycobacterium tuberculosis. Here, we describe a novel function of P27 in the interaction of M. bovis with its natural host cell, the bovine macrophage. We found that a deletion in the *p27-p55* operon impairs the replication of M. bovis in bovine macrophages. Importantly, we show for the first time that M. bovis arrests phagosome maturation in a process that depends on P27. This effect is P27 specific since complementation with wild-type *p27* but not *p55* fully restored the wild-type phenotype of the mutant strain; this indicates that P55 plays no important role during the early events of M. bovis infection. In addition, we also showed that the presence of P27 from M. smegmatis decreases the association of LAMP-3 with bead phagosomes, indicating that P27 itself blocks phagosome-lysosome fusion by modulating the traffic machinery in the cell host.

## INTRODUCTION

Mycobacterium bovis causes tuberculosis in a wide variety of mammals with strong tropism for cattle and eventually humans. This mycobacterial species forms, together with M. tuberculosis and many other pathogenic mycobacterial species, the M. tuberculosis complex (MTC). The members of the MTC are genetically related and may have evolved from a common ancestor via successive DNA deletions/insertions, resulting in the present Mycobacterium speciation and their differences in pathogenicity (1). In recent years, considerable advances have been made in the understanding of the molecular bases of pathogenicity, virulence, and persistence of mycobacteria. One significant contribution has been the identification and characterization of essential mycobacterial virulence genes. Most of these studies have been performed in the human bacillus M. tuberculosis because of its worldwide distribution and its impact on global health. Although the bovine macrophage is the preferred site of infection for M. bovis, little is known about the host-pathogen interactions between M. bovis and the bovine macrophage.

P27 is a secreted surface-expressed glycolipoprotein antigen that was first described in M. bovis and is conserved in many species of the Mycobacterium genus (2). The gene that encodes P27 constitutes an operon, together with the *Mb1445c* gene, usually known as *p55* (2). The mutation of the *p27-p55* operon in both M. bovis and M. tuberculosis significantly reduces the replication of the bacilli in mice and in macrophage cell cultures; this indicates that both P27 and P55 are relevant for mycobacterial virulence (3, 4). The first functional studies of the *p27-p55* operon demonstrated that LprG (P27) participates in host-pathogen adhesion through binding to dendritic cell-specific intercellular adhesion molecule-3-grabbing nonintegrin (DC-SIGN) receptor (5). DC-SIGN receptor, like mannose receptor, is a C-type lectin receptor present in dendritic cells and macrophages. These findings suggest that interactions between these cells and P27 occurred via its mannosylated residues (5). P27 also binds Toll-like receptor 2 (TLR2) (6, 7), an important component of the innate immune response against M. tuberculosis (8) and virulent M. bovis (9). The deletion of the *p27-p55* operon has shown to reduce the capacity of M. bovis to induce an adequate Th1 response in cattle (10), underlining the immunogenic properties of P27. Later on, two studies reported that the proteins encoded in this operon are involved in the preservation of the cell wall and in the transport of toxic compounds away from the cells (4, 11). Recent studies have proposed that the role of the proteins encoded in the *p27*(*lprG*)-*p55* operon is to facilitate the surface localization of mannose-capped lipoarabinomannan (ManLAM) (12, 13). The ManLAM exposed on the bacterial surface binds the mannose receptor on macrophages, allowing the phagocytosis of the mycobacteria and subsequently the survival in arrested phagosomes (14). In this model, the lack of P27 (LprG) would impair the inhibitory mechanism of phagosome-lysosome fusion by reducing the expression of ManLAM in the bacterial surface. Although these previous studies have undoubtedly demonstrated a role of the *lprG-p55* operon in the ManLAM-mediated cell-host interactions, the contribution of each individual gene of the *lprG-p55* operon to these interactions is still undefined. To better understand the mechanism by which P27 and P55 contribute to the virulence of pathogenic mycobacteria, we studied the role of these proteins in the infection by M. bovis of its natural host, the bovine macrophage.

## RESULTS

### Mutation of the *p27-p55* operon impairs M. bovis replication inside bovine macrophages.

**FIG 1:**
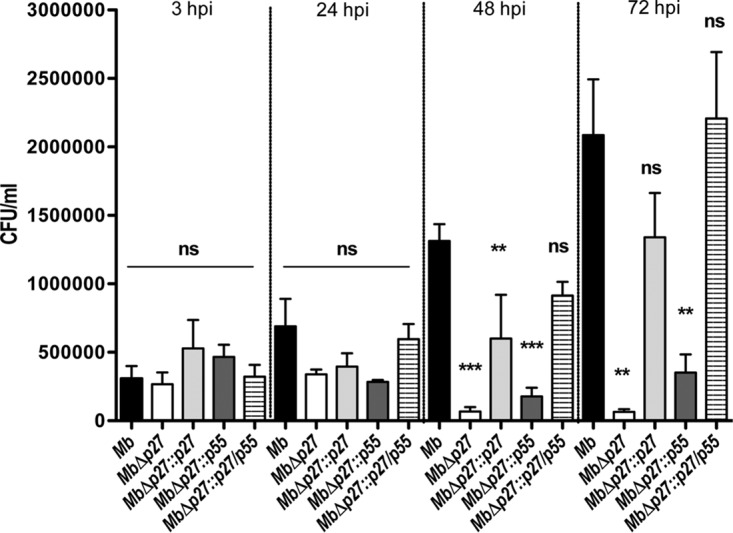
Replication of the MbΔp27 mutant in BMDMs. The bacterial growth in BMDMs assessed by CFU is depicted. Infections were performed with M. bovis wild-type (black bars), mutant MbΔp27 (white bars), or complemented strains: MbΔp27::p27 (light gray bars), MbΔp27::p55 (dark gray bars) or MbΔp27::p27/p55 (striped bars). The data are from a representative experiment and indicate the means and standard deviations (SD) of triplicates. The values at each point were significantly different among strains as determined by ANOVA and Bonferroni's multiple-comparison tests. The data represent means ± the SD (*P* < 0.05). The asterisks indicate significance: ***, *P* < 0.001; **, *P* < 0.01; and *, *P* < 0.05. ns, not statistically significant; hpi, hours postinfection.

We have previously reported that P27 and P55 are essential proteins for full M. bovis virulence in BALB/c mice and M. bovis replication in a murine macrophagic cell line (15). To determine whether this role is conserved in the natural host of M. bovis, the bovine macrophages, we compared the intracellular replication of the mutant to the wild type and the complemented strains in bovine monocyte-derived macrophages (BMDMs). Macrophages were infected at a multiplicity of infection (MOI) of 1, and the bacterial burden was calculated by the recovery of intracellular bacteria at different time points postinfection. The number of intracellular bacteria was similar for wild-type and mutant strains at 3 h postinfection, indicating a similar rate of infection for these strains (Fig. 1). Whereas the bacterial burden in cells infected with either the wild type or the fully complemented strain increased after 48 and 72 h, the replication rate of the MbΔp27 mutant was steady and significantly lower (Fig. 1). This finding demonstrates that *p27-p55* mutation impaired M. bovis replication in bovine macrophages. Notably, the complementation with *p55* alone failed to restore the wild-type phenotype. In contrast, the complementation with *p27* alone enabled the mutant to replicate at levels equivalent to those of a double-complemented or the wild-type strain at 72 h postinfection.

### The *p27-p55* operon is required for M. bovis phagosome maturation arrest in bovine macrophages.

**FIG 2:**
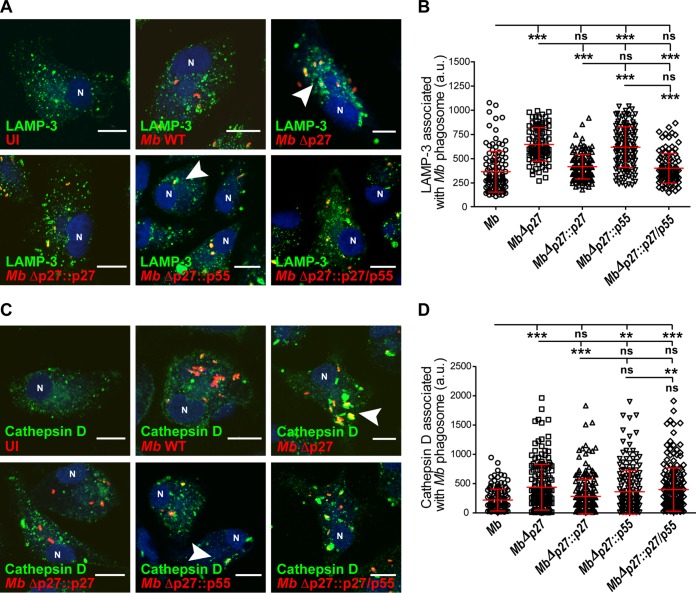
Mutation of the *p27-p55* operon is required for phagosome maturation arrest. (A) BMDMs were infected with the different M. bovis strains labeled with rhodamine (red) during 1 h of uptake and 2 h of chase. The cells were fixed and subjected to indirect immunofluorescence with an antibody against LAMP-3 (green). Nuclei were stained with DAPI (4′,6′-diamidino-2-phenylindole [blue]). The arrowheads in the images show the mycobacterial phagosomes. Scale bars, 10 μm. (B) Quantitation of LAMP-3 associated with M. bovis phagosomes. Results for M. bovis wild type (○), mutant MbΔp27 (□), and complemented strains MbΔp27::p27 (△), MbΔp27::p55 (▽), and MbΔp27::p27/p55 (♢) are shown. (C) BMDMs were infected with the different M. bovis strains labeled with rhodamine (red) for 1 h of uptake and 2 h of chase. The cells were fixed and subjected to indirect immunofluorescence with an antibody against cathepsin D (green). Nuclei were stained with DAPI (blue). The arrowheads in the merge images show the mycobacterial phagosomes. Scale bars, 10 μm. (D) Quantitation of cathepsin D associated with M. bovis phagosomes. Results for M. bovis wild type (○), mutant MbΔp27 (□), and complemented strains MbΔp27::p27 (△), MbΔp27::p55 (▽), and MbΔp27::p27/p55 (♢) are shown. The cells were analyzed by confocal microscopy and quantified using Fiji software. The data represent the means ± the standard errors of the mean (SEM) of three independent experiments. The values at each point were significantly different among strains, as determined by ANOVA and Tukey's multiple-comparison tests. The asterisks indicate significance: ***, *P* < 0.001; **, *P* < 0.01; and *, *P* < 0.05. ns, not statistically significant.

To further understand the mechanism by which P27-P55 counteracts the microbicidal actions of the bovine macrophages, we evaluated the maturation stage of mycobacterial phagosomes by using immunofluorescence and confocal microscopy. M. bovis strains were used to infect bovine macrophages during 1 h of uptake and 2 h of chase, as described in Materials and Methods. M. bovis wild-type association with the late endocytic marker lysosomal-associated membrane protein 3 (LAMP-3) was relatively low. In contrast, the fraction of MbΔp27 associated with LAMP-3 was significantly higher (*P* ≤ 0.05) than that of the wild-type strain (Fig. 2A and B). These results indicate that the *p27-p55* operon participates in the phagosomal arrest induced by intracellular M. bovis to replicate inside macrophages. To assess the contribution of each protein of the *p27-p55* operon to the phagosome arrest, we evaluated the association of LAMP-3 with phagosomes containing MbΔp27 complemented with the *p27* or *p55* gene of the operon. After infection, the association of LAMP-3 with MbΔp27::p55 was not significantly different from the MbΔp27 strain. Conversely, the association of LAMP-3 with MbΔp27::p27 was lower than that for infections with MbΔp27 and equivalent to those for infections with either with the double-complemented MbΔp27::p27/p55 strain or the wild-type strain. To confirm these results, we additionally analyzed the association of the lysosomal enzyme cathepsin D. Similarly, the association fraction of cathepsin D with mycobacteria was higher in bovine macrophages infected with MbΔp27 than in those infected with either the wild-type or the MbΔp27::p27 strain (Fig. 2C and D). Altogether, we concluded that the M. bovis-induced phagosomal arrest in bovine macrophages required P27.

### P27 blocks polystyrene bead-phagosome (BP) maturation.

**FIG 3:**
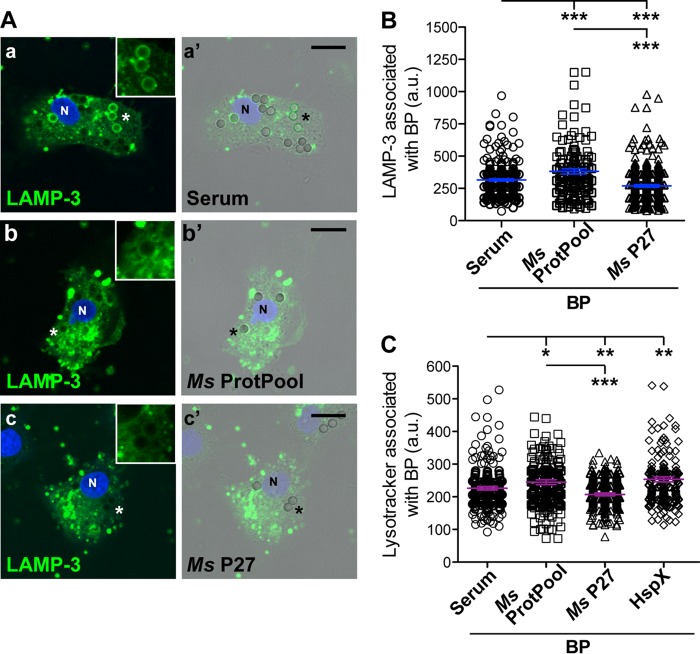
P27 decreases the association of LAMP-3 with polystyrene bead-phagosomes (BPs). (A) BMDMs were incubated with 3-μm polystyrene beads coated with bovine serum proteins (serum images a and a′), a pool of M. smegmatis proteins (ProtPool images b and b′), or M. smegmatis P27 protein (P27 c and c′) for 1 h of uptake. Next, the cells were washed and incubated for 1 h of chase. Subsequently, the cells were fixed and stained with anti-LAMP-3 antibody, followed by Alexa Fluor 488-coupled anti-goat IgG (green), and then analyzed by confocal microscopy. Phase-contrast images show the beads. Nuclei were stained with DAPI (blue). Scale bars, 10 μm. Insets show the BPs labeled with asterisks in the cells. (B) Quantitative analysis of the LAMP-3 fluorescence intensity association to polystyrene bead-phagosomes (BPs) coated with the different proteins shown in panel A. (C) BMDMs were incubated with 3-μm polystyrene beads coated with bovine serum proteins (serum), a pool of M. smegmatis proteins (ProtPool), M. smegmatis P27 protein (P27), or HpsX protein for 1 h of uptake. Next, the cells were washed, followed by incubation for 1 h of chase with the addition of the dye LysoTracker Red (50 nM) to detect acidic compartments. The cells were fixed and analyzed by confocal microscopy. The fluorescence intensity association of LysoTracker to the BPs was quantified. Data represent the means ± the SEM of three independent experiments. In all panels, the asterisks indicate significance: *, *P* ≤ 0.05; **, *P* ≤ 0.01; and ***, *P* ≤ 0.001. The data were analyzed using a two-tailed Student *t* test.

The results described above indicate that only P27, and not P55, seems to be necessary for limiting phagosome-lysosome fusion, suggesting that the function of the P27-P55 system is not involved in the phagosome arresting mechanism. In order to test whether P27 itself influences phagosome maturation, we determined the maturation stage of phagosome-containing P27 in a bacterium-free context. It has been reported that P27 captures in its hydrophobic pocket and in other protein domains, LAM, lipomannan (LM) and other glycolipids, particularly phosphatidyl-myoinositol mannosides (PIM) (6). Mannose-capped LAM (ManLAM) is a key component of the interaction between pathogenic mycobacterium and host cells (16), and this complex glycolipid is not produced by M. smegmatis. Instead, M. smegmatis produces phospho-myoinositol-capped LAM (PI-LAM) and PIM (17). To determine whether the role of P27 in host cell interaction is dependent on ManLAM, P27 was purified from M. smegmatis as a recombinant hexahistidine-tagged form. Recombinant P27 was absorbed onto polystyrene beads, and the presence of the protein on coated beads was verified by Western blotting (see Fig. S1 in the supplemental material). As a control for the phagocytosis of coated beads, we used bovine serum proteins, recombinant HspX (a heat shock protein not associated with phagosome maturation [purified from Escherichia coli]), and a pool of M. smegmatis protein debris (ProtPool) that binds nonspecifically to the nickel-resin (see Materials and Methods). Coated beads were incubated with BMDMs and, upon phagocytosis, the trafficking of bead-phagosomes (BPs) was analyzed using LAMP-3 and LysoTracker (LTR), a fluorescent dye used for labeling acidic bead-phagosomes (18), and then analyzed by confocal microscopy (Fig. 3). Our results showed that the presence of P27 from M. smegmatis decreased the association of LAMP-3 with bead-phagosomes (Fig. 3A and B). In contrast, the LAMP-3 association with ProtPool-coated beads markedly increased, indicating that P27 is sufficient to arrest phagosome maturation of latex bead-phagosomes. Confirming this result, the association of LTR with P27-coated BPs significantly decreased in bovine macrophages (Fig. 3B).

### P27 increases the survival of M. bovis in bovine macrophages and HeLa cells.

**FIG 4:**
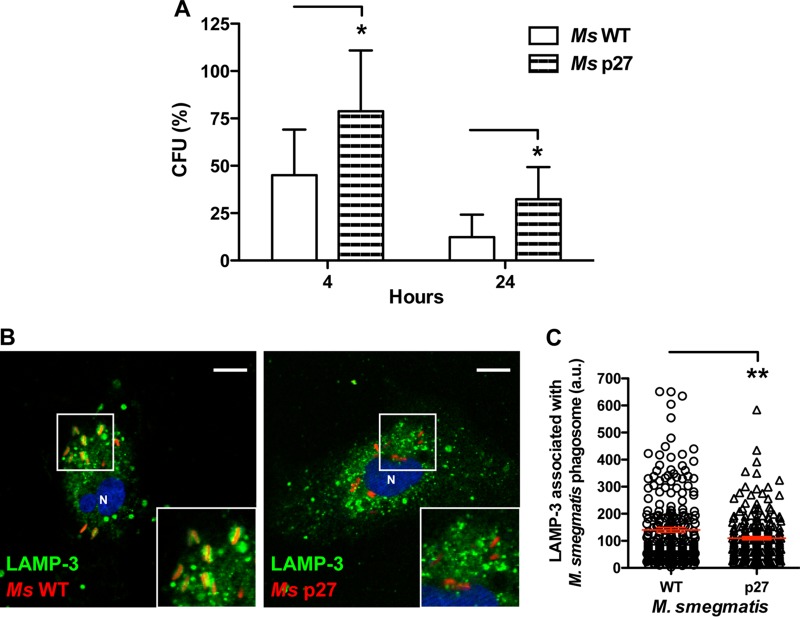
P27 favors the survival of M. smegmatis and impairs the phagosome maturation in bovine macrophages. (A) M. smegmatis survival in BMDMs assessed by CFU is shown. Infections were performed with M. smegmatis wild type (white bars) or M. smegmatis overexpressing P27 (striped bars) for 4 and 24 h. Data represent the means ± the SD of three independent experiments. Asterisks indicate significance (*, *P* ≤ 0.05). (B) BMDMs were infected with M. smegmatis wild type and M. smegmatis overexpressing P27, both labeled with rhodamine (red), for 1 h of uptake, followed by 1 h of chase. The cells were fixed and subjected to indirect immunofluorescence with an antibody to LAMP-3 (green). Scale bars, 10 μm. The inset shows the M. smegmatis phagosomes associated with LAMP-3. (C) Quantitative analysis of the LAMP-3 association with M. smegmatis wild type or M. smegmatis overexpressing P27-containing phagosomes in BMDMs after 2 h of infection. The data represent means ± the SEM of three independent experiments (**, *P* ≤ 0.01 [two-tailed Student *t* test]).

To determine the importance of P27 in the mycobacterial intracellular persistence, we infected bovine macrophages with M. smegmatis wild type or M. smegmatis strains overexpressing P27. After 4 and 24 h of infection, we observed a significant increase in the survival of M. smegmatis expressing P27 (Fig. 4A), although the number of bacteria of both strains decreased between 4 and 24 h. Although the bacteria are eliminated in bovine macrophages, the enforced expression of P27 has a negative effect in the killing of the bacteria. In addition, when we analyzed the association of LAMP-3 with the mycobacterial phagosomes after 3 h of infection, the LAMP-3 association with M. smegmatis overexpressing P27 markedly decreased (Fig. 4B and C) compared to the wild-type strain. Thus, P27 affects the maturation of mycobacterial phagosomes, followed by the increase in the survival of M. smegmatis in bovine macrophages at early postinfection times. We next evaluated the survival of M. smegmatis wild type and an M. smegmatis strain overexpressing P27 in HeLa cells (see Fig. S2 in the supplemental material). We observed an increase in the survival of the P27-overexpressing strain after 24 and 48 h of infection, indicating that P27 also increases the intracellular persistence of M. smegmatis in nonprofessional phagocytes. In addition, these results confirm that the role of P27 in arresting phagosome maturation of bovine macrophages does not require P55.

### Mutation of the *p27-p55* operon produces low expression of iNOS.

**FIG 5:**
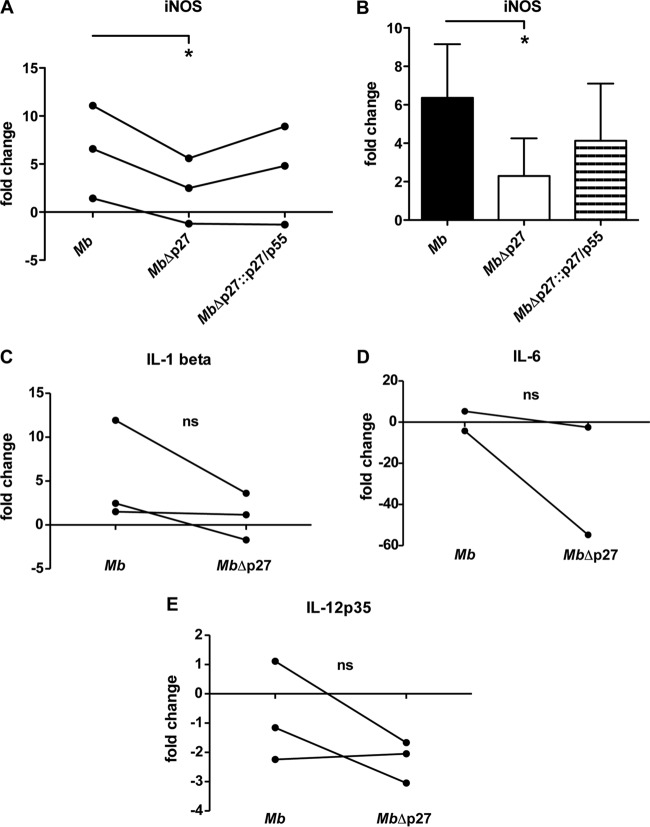
Relative iNOS and cytokine expression in BMDMs. (A and B) Gene expression of iNOS measured in BMDMs infected with M. bovis wild type, mutant MbΔp27, or complemented strain MbΔp27::p27/p55. The relative gene expression was calculated using the 2^−ΔΔ*CT*^ method with E correction, using GAPDH mRNA expression as the reference gene and uninfected cells as the calibrator. Asterisks indicate significance (*, *P* ≤ 0.05). The data were analyzed using a paired Student *t* test. (A) Replicates of the experiments. (B) Data expressed as a bar graph. (C, D, and E) Gene expression was measured in BMDMs infected with M. bovis wild type or mutant MbΔp27. The relative gene expression of IL-1β (C), IL-6 (D), or IL-12p35 (E) was calculated using the 2^−ΔΔ*CT*^ method with E correction, using GAPDH mRNA expression as the reference gene and uninfected cells as the calibrator. Data were analyzed using a paired Student *t* test. ns, not statistically significant.

In addition to phagosome maturation, nitric oxide (NO) production is a mechanism that controls mycobacterial replication. To explore the capacity of the mutant MbΔp27 to induce proinflammatory response in bovine macrophages, we evaluated the expression of cytokines and inducible nitric oxide synthase (iNOS) upon infection. The MbΔp27 strain elicited significantly lower transcript levels of iNOS at 16 h postinfection than the wild-type strain (Fig. 5A and B). However, infection with the complemented strain MbΔp27::p27/p55 produced a partial recovery of iNOS transcription. Despite the upregulation of iNOS upon M. bovis infection, the production of NO in culture supernatants of infected bovine macrophages was undetectable (data not shown). This result may be because of the lack of interferon gamma stimulation during the cell culturing, which is a well-known enhancer of NO production (19, 20).

Although not significant, the expression of the proinflammatory cytokines interleukin-1β (IL-1β), IL-6, and IL-12p35 showed a reduction trend in macrophages infected with the mutant strain compared to those infected with the wild-type strain (Fig. 5C, D, and E).

## DISCUSSION

A hallmark of pathogenic mycobacteria is the lack of classical virulence factors that are present in many other bacterial pathogens. In nonpathogenic mycobacteria in particular, many virulence genes, such as P27/LprG, are conserved. These findings suggest that pathogenic genomes have adapted their free lifestyle to the intracellular environment with minimal acquisition of new or exclusive virulence genes. Many of the known virulence factors are needed for the survival of bacilli inside macrophages. These particular virulence factors act by modulating or blocking the defense mechanisms that the host cell displays to eliminate mycobacteria. For instance, the production of effectors, including those involved in responses to nitro-oxidative stress and cell death program or apoptosis, is a clear example of these mechanisms.

An effective strategy used by M. tuberculosis to counteract the action of macrophages is the subversion of the normal progression of phagosome maturation to prevent the formation of an active phagolysosome. This modulation of intracellular endosomal trafficking allows the bacteria to remain in a replicative niche and thus avoid immune detection. Although the process of phagosomal arrest induced by M. tuberculosis is well described, this cell-autonomous mechanism of defense is less clear in M. bovis. Moreover, at longer time points after infection (e.g., 7 days), M. bovis is able to translocate into the cytosol of human cells (21), although whether M. bovis is localized in the cytosol of bovine macrophages remains to be determined. Two previous studies (12, 13) proposed that LprG-P55 serves as a carrier to facilitate assembly or trafficking of ManLAM to the M. tuberculosis cell wall, which in turn binds to the macrophage-mannose receptor and facilitates bacterial entry and the inhibition of phagosome-lysosome fusion. Furthermore, ManLAM induces iNOS through the NF-κB signaling pathway (22). Therefore, it is possible that the prominent localization of the mutant MbΔp27-p55 in phagolysosomes, as well as the significant decrease in the levels of iNOS induced by this mutant compared to the wild-type strain, is a consequence of lower ManLAM levels on the cell surface, leading to a reduction in the survival of the mutant in macrophages.

This study highlights that P55 is dispensable for the inhibition of phagosome-lysosome fusion since the M. bovis mutant expressing P27 alone restored the wild-type intracellular persistence at 72 h postinfection and was capable of inducing phagosome arrest at the same level as that of the wild-type strain. These results suggest that P27 does not require P55 to facilitate ManLAM localization on the bacterial surface, that P27 inhibits phagosome-lysosome fusion by direct binding to the mannose receptor via its mannose residues, or both. The fact that recombinant P27 purified from M. smegmatis inhibited the maturation of bead-containing phagosomes to phagolysosomes suggests that P27 exerts a direct interaction with the host cells, which does not preclude its previously described function in the cell wall assembly. Moreover, the localization of P27 in the extracellular space or in association with the bacterial cell wall (15) also supports a model in which P27 can trigger the blockage of phagosome-lysosome fusion by direct binding to the mannose receptor. We hypothesize that this binding to the mannose receptor is via its associated glycolipids, likely PIM_6_ (17).

In summary, we characterized here for the first time the trafficking of M. bovis phagosomes in a primary culture of bovine macrophages. This study demonstrates that virulent M. bovis arrests phagosome maturation in bovine macrophages and that P27 plays a key role in this mechanism. Importantly, although both P27 and P55 are necessary for M. bovis replication inside both bovine and mouse macrophages (15), P27 alone has an additional role in the mycobacterial survival inside bovine macrophages and in nonprofessional phagocytes. Therefore, our results, together with previous reports (12, 13, 15), indicate that the *p27-p55* operon is involved in multiple virulence mechanisms and functions and that P27 plays a major role in the early events of M. bovis infection (5, 6). The precise mechanism by which P27 modulates M. bovis trafficking is still unknown, as well as the host cell factors subverted by this virulence factor. Our findings do demonstrate that this protein directly participates in one important survival mechanism displayed by pathogenic mycobacteria. It remains unclear, however, whether there is any cooperative action between LprG/P27 and P55 in the transfer of ManLAM and glycolipids to the bacterial cell envelope.

## MATERIALS AND METHODS

### Bacterial strains and culture media.

The M. bovis strains were grown in Middlebrook 7H9 or Middlebrook 7H10 medium (Difco Laboratories, USA) supplemented with 0.5% albumin, 0.4% dextrose, and 0.5% pyruvate. Middlebrook 7H9 was used with or without 0.05% Tween 80. When necessary, either hygromycin at 50 μg/ml or kanamycin at 20 μg/ml (Sigma, USA) was added to the media.

### PBMC isolation and BMDM differentiation.

The animals used in this study were selected from the Instituto Nacional de Tecnologías Agropecuarias (INTA) experimental herd and tested negative for bovine tuberculosis infection for the single intradermal tuberculin test and interferon gamma release test. Portions (60 ml) of blood were taken from each animal under sterile conditions according to the instructions of the Committee for Institutional Care and Use of Animal Experimentation (CICUAE-CICVyA) of INTA. Peripheral blood mononuclear cells (PBMCs) were separated from heparinized blood by centrifugation over Histopaque 1077 (Sigma) according to the manufacturer's protocol. To derive monocytes, we cultured PBMCs on 12-mm glass coverslips, T25 flasks, or 24-well plates containing RPMI 1640 complete medium (Invitrogen, Carlsbad, CA) supplemented with 10% autologous plasma for 16 h at 37°C and 5% CO_2_. Nonadherent cells were removed by extensive washing with phosphate-buffered saline (PBS). Only adherent cells were maintained in culture for 5 days at 37°C and 5% CO_2_ to obtain BMDMs. Cell viability was confirmed by trypan blue exclusion assay.

### BMDM infections.

M. bovis was cultured until exponential growth phase, harvested, washed to eliminate the rest of the bacterial culture medium, and then resuspended in RPMI medium. The bacterial suspensions were passed through a syringe needle (25 gauge) to disaggregate bacterial clumps. The remaining clumps were removed by ultrasonic treatment in a water bath, followed by a low-speed centrifugation for 5 min. The optical densities of clump-free bacterial suspensions were adjusted to MOIs of 10, 5, or 1 according to the experiment. The MOIs were 10 for the intracellular trafficking assays, 5 for the isolation of RNA for the reverse transcription-quantitative PCR (RT-qPCR) assay, and 1 for the bacterial replication assays. The BMDMs were infected for 1 h at 37°C and 5% CO_2_ (uptake) and then were washed three times to eliminate the extracellular bacteria. Subsequently, BMDMs were incubated for another 2 or 16 h or different time points (chase) for intracellular trafficking, RNA isolation, or bacterial replication assays, respectively. Three independent infections were performed for each assay. The experiments with M. smegmatis strains were performed in the same way.

### Indirect immunofluorescence and confocal microscopy.

M. bovis or M. smegmatis strains treated as described above were covalently stained with rhodamine 5(6)-carboxytetramethylrhodamine *N*-succinimidyl ester (Sigma, Germany). Briefly, 10^9^ bacteria were washed twice with 0.1 M PBS and suspended in 1 ml of the same solution. Rhodamine was added to a final concentration of 5 μg/ml, followed by incubation in the dark for 1 h at 37°C. The bacteria were next washed gently with PBS until unbound colorant was eliminated and then used to infect BMDMs as described above. The infected cells were fixed with 4% paraformaldehyde solution in PBS (PFA) for 20 min and quenched by incubation with 50 mM glycine solution for 30 min. The cells were then permeabilized with 0.05% saponin in PBS containing 1% bovine serum albumin (BSA) for 15 min, followed by incubation with the corresponding primary antibody diluted 1:50 in PBS. To localize host bovine proteins, we tested different antibodies for several markers. Of the antibodies tested, only LAMP-3 (Santa Cruz Biotechnology, Santa Cruz, CA) and cathepsin D (Santa Cruz Biotechnology) were labeling specific structures in bovine macrophages. Secondary anti-goat antibodies conjugated to Alexa 488 (Jackson Immuno Research Labs, Inc.) were used diluted 1:500 in PBS. Each incubation step with the antibodies lasted 1.5 h. To detect acidic compartments, the dye LysoTracker Red (Invitrogen) was used. The cells were incubated with 50 nM LysoTracker at 37°C for 1 h. The cells were mounted with mounting medium (Dako, Denmark) and analyzed by confocal microscopy using an SP5 AOBS confocal microscope (Leica Microsystems, Germany). Mycobacterial internalization was monitored using the fluorescence of red rhodamine, and the LAMP-3 or cathepsin D association with mycobacterial phagosomes was analyzed in at least 100 cells using Fiji software (U.S. National Institutes of Health, Bethesda, MD) as described previously (18). Experiments were performed in duplicates in three independent experiments. Statistical analysis was performed using analysis of variance (ANOVA) and Bonferroni's multiple-comparison tests.

### Isolation of RNA from macrophages.

At 16 h after infection, the cells were lysed with 1 ml of chilled TRIzol (Invitrogen). TRIzol was removed from the flasks, and the lysate was homogenized. Cellular RNA extractions were then performed according to the manufacturer's instructions for TRIzol reagent (Invitrogen). The RNA pellets were purified by precipitation with LiCl (Ambion, USA) and resuspended in 30 μl of RNase-free water. Total RNA quality and quantity were analyzed by spectrophotometry (NanoDrop, Wilmington, DE) and electrophoresis on a 0.8% agarose gel.

### RT-qPCR.

DNA-free RNA (1 μg) was mixed with 50 ng of random primers (Invitrogen) in a 20-μl final volume and reverse transcribed to total cDNA with SuperScript II reverse transcriptase (Invitrogen) according to the manufacturer's instructions. Approximately 25 ng of the cDNA was used as the template for each real-time qPCR analysis. qPCRs were performed on an Applied Biosystem Step One Plus instrument using *Taq* platinum, SYBR green I dye (Invitrogen), and specific primers (Table 1) under standard cycling conditions. All reactions were performed in triplicates and qPCR data were analyzed using LinReg (23). Fg statistical software (24) was used for curve analysis and ratio calculation. To assess differences on gene expression in macrophages within groups, we used GAPDH (glyceraldehyde-3-phosphate dehydrogenase) as the control gene and data from noninfected BMDMs as the calibrator (25).

**TABLE 1:** Primer sequences used for PCR experiments

Primer^*a*^	Sequence (5′–3′)
GAPDH f	ATCTCTGCACCTTCTGCCGA
GAPDH r	GCAGGAGGCATTGCTGACA
IL-12p35 f	TAGCCACGAATGAGAGTTGCC
IL-12p35 r	TTTCCAGAAGCCAGACAATGC
IL-1b f	TGACCTGAGGAGCATCCTTT
IL-1b r	CCAGCGATTTTTGCTCTCTG
IL-6 f	TGCTTGATCAGAACCACTGC
IL-6 r	GCGATCTTTTGCTTCAGGAT
iNOS f	AGAGCCTCTGGACCTCAACA
iNOS r	CTGCCCTCACAGGAGAGTTC

a f, forward; r, reverse.

### Protein purification of M. smegmatis proteins.

P27 was purified as fusion to six histidines from M. smegmatis mc^2^155 carrying plasmid pW16-Rv1411c (Bei Resources). Briefly, 100 ml of M. smegmatis strains was pelleted by centrifugation and resuspended in 2 ml of lysis buffer (50 mM NaH_2_PO_4_, 300 mM NaCl, 10 mM imidazole [pH 8.0]). Cells were disrupted in a Precellys cell homogenizer (Bertin Instruments, France). Soluble proteins were incubated with 200 μl of nickel-charged resin (Qiagen, Hilden, Germany) and purified according to the manufacturer's recommendations. Purified proteins were dialyzed against 0.1 M borate buffer (pH 8.5) overnight at 4°C.

### Bead preparation.

Polystyrene beads (3 μm; Krisker Biotech, Steinfurt, Germany) were coupled with 20-μg/ml concentrations of the following proteins: bovine serum proteins (serum), a pool of proteins of M. smegmatis (ProtPool), or M. smegmatis P27 protein (P27). The coupling was performed using 0.1 M borate buffer (pH 8.5) at room temperature overnight on a rotating wheel. The beads were washed with BSA at 10 mg/ml in borate buffer and finally resuspended in PBS (pH 7.4) containing BSA at 10 mg/ml and stored at 4°C until use.

### SDS-PAGE and Western blot analysis.

To detect the adsorption of P27 to the polystyrene beads, beads coated with bovine serum proteins (serum), a pool of proteins of M. smegmatis (ProtPool), or the P27 protein of M. smegmatis (P27) as described above were resuspended in sample buffer containing 1% 2-mercaptoethanol, and the samples were maintained at 4°C. For the Western blot analyses, the samples were subjected to electrophoresis in a 12% SDS-PAGE gel, transferred to a nitrocellulose membrane, and blocked with PBS supplemented with 0.1% (vol/vol) Tween 20 and 5% milk. The nitrocellulose membrane was incubated for 2 h with a polyclonal anti-P27 antibody, washed, and incubated with a secondary alkaline phosphatase-conjugated goat anti-rabbit antibody (Sigma, USA) at a 1:10,000 dilution.

### Internalization of polystyrene beads.

For internalization, coated beads were diluted 1:500 in complete medium and applied to PBMC-derived bovine macrophages seeded in a 24-well plate with a final concentration of ∼10 beads per cell. After the indicated times of uptake and chase, the cells were washed with PBS and fixed for 20 min with 4% PFA.

### Image analysis.

A Leica SP5 AOBS laser scanning confocal microscope was used (Leica Microsystems, Germany). For image acquisitions in fixed samples, a single focal plane was monitored over time (xyt scanning mode) using a 63×/1.4 HCX-PLAPO oil objective lens, an argon laser (488 nm), and a DPSS laser (561 nm), when applicable; a scanner frequency of 200 to 400 Hz; and line averaging 6, using PMT detectors at a scanning resolution of 1,024 by 1,024 pixels or 512 by 512 pixels (zoom of 2.5). The same settings for laser powers, gain, and offset were maintained for the different experiments. However, they were not obtained close together in time. The microscope software (LAS AF) permits saving the exact settings to acquire the images used in the experiments. Therefore, with every experiment the corresponding settings were loaded.

Analyses of all the images were performed using ImageJ (National Institutes of Health) and Fiji. Fiji is a distribution of ImageJ available at http://fiji.sc. Iterative versions of ImageJ used for this work are 1.41m through 1.46a.

To measure the association of a marker (e.g., primary antibodies against LAMP-3 or cathepsin D) with bacterial particles in the cell, the RGB image was split into individual channels, and the red channel (bacteria) was subjected to a pixel threshold. The “wand-tracing tool” was used to select all the bacteria per cell, and then the “analyzed-measure” function of Fiji was used to measure the fluorescence intensity of the marker of interest (corresponding to the secondary antibody used to detect the primary antibody) associated with the bacterial phagosome by redirecting the measurements to the channel of interest in “set measurements” in the analysis function of Fiji.

To quantify the fluorescence association with the bead phagosomes, the corresponding fluorescent-channel movies were loaded into Fiji. A circle enclosing the phagosome in the bright-field image was drawn by using the “elliptical selection” tool. In “set measurements,” only “area” and “integrated density” were selected. Subsequently, the fluorescence intensity of the marker of interest (corresponding to primary antibodies against LAMP-3 or LysoTracker) associated with the bead phagosome was measured by redirecting the measurements to the channel of interest in “set measurements” in the analysis function of Fiji (18). All of the images were acquired with the same zoom, and the results of the different experiments were combined. The fluorescence intensity values were plotted and analyzed using Microsoft Excel 2011 (Microsoft) and GraphPad Prism 5 (GraphPad Software, Inc., USA).

## Supplementary Material

Supplemental material
