# Supplementary material for: Mycobacterium bovis Requires P27 (LprG) To Arrest Phagosome Maturation and Replicate within Bovine Macrophages
Source: Infect Immun. 2017 Feb 23;85(3):e00720-16. doi: 10.1128/IAI.00720-16 (PMC5328499; doi:10.1128/IAI.00720-16)
Supplement: Supplemental material [file supp_85_3_e00720-16__index.html]

Supplemental material 

# Mycobacterium bovis Requires P27 (LprG) To Arrest Phagosome Maturation and Replicate within Bovine Macrophages

## Supplemental material

- Supplemental file 1 -

  Fig. S1. Detection of P27 protein on polystyrene beads.

  PDF, 4.6M
- Supplemental file 2 -

  Fig. S2. Survival of *M. smegmatis* wild type and the strain overexpressing P27 in HeLa cells.

  PDF, 453K
