# Supplementary material for: Mycobacterium bovis Requires P27 (LprG) To Arrest Phagosome Maturation and Replicate within Bovine Macrophages
Source: Infect Immun. 2017 Feb 23;85(3):e00720-16. doi: 10.1128/IAI.00720-16 (PMC5328499; doi:10.1128/IAI.00720-16)
Supplement: Supplemental material [file IAI.00720-16_zii999091974s1.pdf]

Supplementary Figure 1

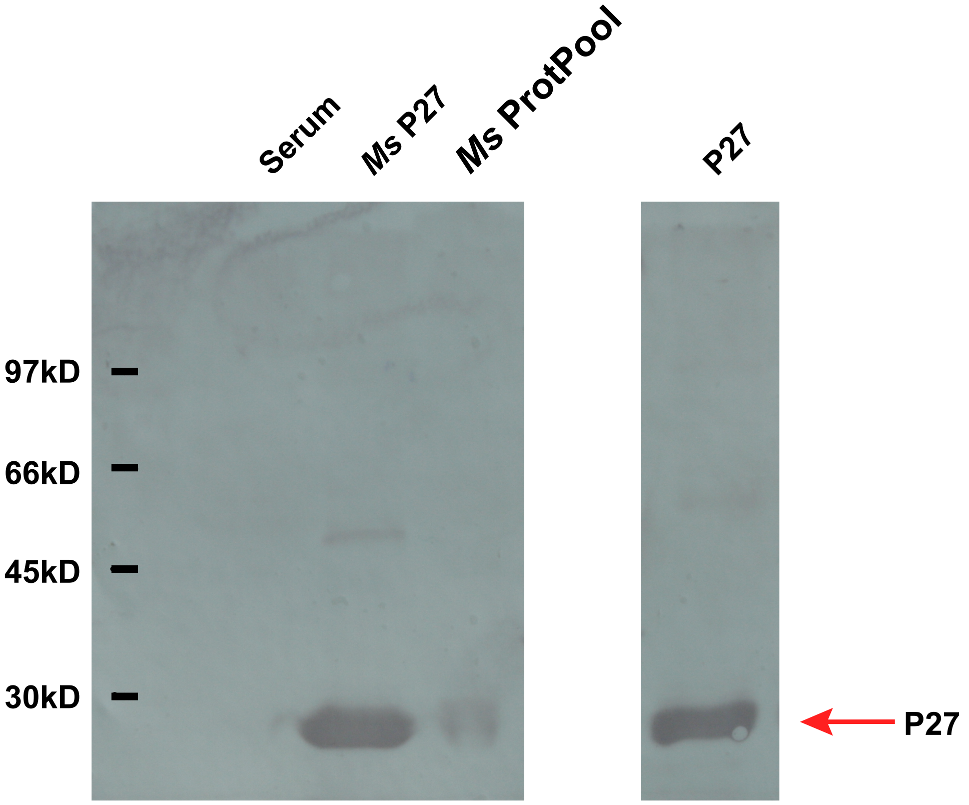

Supp. Figure 1: Detection of P27 protein coated to polystyrene beads. Image shows the immunoblots from the polystyrene beads coated with bovine serum proteins (Serum), a pool of proteins of *M. smegmatis* (*Ms* ProtPool) or P27 protein of *M. smegmatis* (*Ms* P27). Adsorption of P27 to the polystyrene beads was detected using polyclonal anti-P27 antibody.
