# Supplementary material for: Mycobacterium bovis Requires P27 (LprG) To Arrest Phagosome Maturation and Replicate within Bovine Macrophages
Source: Infect Immun. 2017 Feb 23;85(3):e00720-16. doi: 10.1128/IAI.00720-16 (PMC5328499; doi:10.1128/IAI.00720-16)
Supplement: Supplemental material [file IAI.00720-16_zii999091974s2.pdf]

Supplementary Figure 2

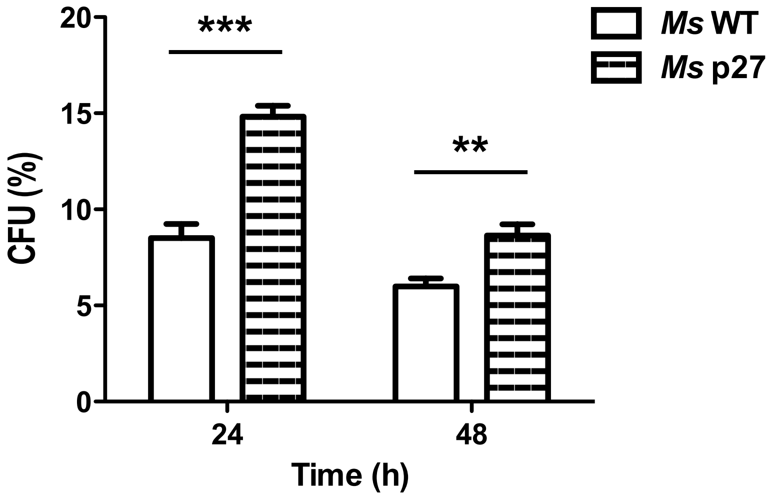

Supp. Figure 2: Survival of *M. smegmatis* wild type and the strain overexpressing P27 in HeLa cells. HeLa cells were infected with *M. smegmatis* wild type and *M. smegmatis* overexpressing P27 (*Ms* p27) for 1 h of uptake, followed by 24 h and 48 h of chase. Then cells were lysed and CFU determined. Data represent the Mean ± S.E.M of three independent experiments. Asterisks show significance (**) $p \leq 0.01$, (***) $p \leq 0.001$ using two-tailed Student's t-test.
